# Effects of manual hyperinflation in preterm newborns under mechanical ventilation

**DOI:** 10.5935/0103-507X.20160058

**Published:** 2016

**Authors:** Camila Chaves Viana, Carla Marques Nicolau, Regina Celia Turola Passos Juliani, Werther Brunow de Carvalho, Vera Lucia Jornada Krebs

**Affiliations:** 1 Instituto da Criança, Hospital das Clínicas, Faculdade de Medicina, Universidade de São Paulo - São Paulo (SP), Brazil.; 2 Department of Pediatrics, Hospital das Clínicas, Faculdade de Medicina, Universidade de São Paulo - São Paulo (SP), Brazil.

**Keywords:** Respiratory therapy, Respiration, artificial, Positive-pressure respiration, Infant, newborn, Intensive care, neonatal

## Abstract

**Objective:**

To assess the effects of manual hyperinflation, performed with a manual resuscitator with and without the positive end-expiratory pressure valve, on the respiratory function of preterm newborns under mechanical ventilation.

**Methods:**

Cross-sectional study of hemodynamically stable preterm newborns with gestational age of less than 32 weeks, under mechanical ventilation and dependent on it at 28 days of life. Manual hyperinflation was applied randomly, alternating the use or not of the positive end-expiratory pressure valve, followed by tracheal aspiration for ending the maneuver. For nominal data, the two-tailed Wilcoxon test was applied at the 5% significance level and 80% power.

**Results:**

Twenty-eight preterm newborns, with an average birth weight of 1,005.71 ± 372.16g, an average gestational age of 28.90 ± 1.79 weeks, an average corrected age of 33.26 ± 1.78 weeks, and an average mechanical ventilation time of 29.5 (15 - 53) days, were studied. Increases in inspiratory and expiratory volumes occurred between time-points A5 (before the maneuver) and C1 (immediately after tracheal aspiration) in both the maneuver with the valve (p = 0.001 and p = 0.009) and without the valve (p = 0.026 and p = 0.001), respectively. There was also an increase in expiratory resistance between time-points A5 and C1 (p = 0.044).

**Conclusion:**

Lung volumes increased when performing the maneuver with and without the valve, with a significant difference in the first minute after aspiration. There was a significant difference in expiratory resistance between the time-points A5 (before the maneuver) and C1 (immediately after tracheal aspiration) in the first minute after aspiration within each maneuver.

## Abstract (pt)

**Objetivo:**

Avaliar as repercussões da hiperinsuflação manual, realizada com ressuscitador manual com e sem válvula de pressão positiva expiratória final, sobre a função respiratória de recém-nascidos pré-termo em ventilação mecânica.

**Métodos:**

Estudo transversal com recém-nascidos pré-termo com idade gestacional inferior a 32 semanas, em ventilação mecânica e dependentes desta aos 28 dias de vida, estáveis hemodinamicamente. A hiperinsuflação manual foi aplicada de forma randomizada, alternando o uso ou não uso da válvula de pressão positiva expiratória final, seguida de aspiração intratraqueal finalizando a manobra. Para os dados nominais, foi aplicado o teste de Wilcoxon com hipótese bilateral ao nível de significância de 5% e poder de teste de 80%.

**Resultados:**

Foram estudados 28 recém-nascidos pré-termo com peso médio de nascimento 1.005,71 ± 372.16g, idade gestacional média 28,90 ± 1,79 semanas, idade corrigida média de 33,26 ± 1,78 semanas, tempo médio de ventilação mecânica de 29,5 (15 - 53) dias. Ocorreu aumento dos volumes inspiratório e expiratório entre os momentos A5 (antes da manobra) e C1 (imediatamente após aspiração intratraqueal) tanto na manobra com válvula (p = 0,001 e p = 0,009) como sem válvula (p = 0,026 e p = 0,001), respectivamente. Também houve aumento da resistência expiratória entre os momentos A5 e C1 com p = 0,044.

**Conclusão:**

Os volumes pulmonares aumentaram na manobra com e sem válvula, havendo diferença significativa no primeiro minuto após a aspiração. Houve diferença significativa na resistência expiratória entre os momentos A5 (antes da manobra) e C1 (imediatamente após aspiração intratraqueal) no primeiro minuto após a aspiração dentro de cada manobra.

## INTRODUCTION

Improved perinatal care, the use of antenatal corticosteroids, administration of exogenous surfactant and new ventilatory strategies have contributed to the increased survival of increasingly premature children and those with very low birth weight, thereby increasing the number of preterm newborns needing mechanical ventilation.^(1-3)^

Patients under mechanical ventilation present functional changes in the mucociliary system caused by a set of determinant factors, such as the presence of a tracheal tube, the use of high oxygen concentrations, lower airway injury induced by tracheal aspiration and inadequate humidification of the mechanical ventilation system.^(4,5)^ Excessive mucus production, together with the abovementioned factors, increases the risk of sputum retention, lung infection and atelectasis, which are common complications in patients under invasive respiratory support. The impairment of oxygenation due to an intrapulmonary shunt, changes in the ventilation-perfusion ratio, and increased pulmonary vascular resistance can cause lung injury. Sputum retention in the airway provides an ideal environment for colonizing microorganisms, resulting in pneumonia, and a consequent reduction in lung compliance.^(4-6)^

The physical therapy of patients under mechanical ventilation admitted to intensive care units (ICU) includes respiratory physical therapy procedures such as postural drainage, chest vibration, cough, tracheal aspiration and manual hyperinflation (MH), aiming to decrease sputum retention, in order to prevent its complications, to improve oxygenation and to promote the expansion of collapsed areas.^(7-9)^

The application of physical therapy techniques can provide hemodynamic and respiratory stability to these patients. The use of respiratory physical therapy techniques that cause acceptable hemodynamic changes with prolonged pulmonary effects is highly desirable, but more studies are needed in this specific population for improving the approach and treatment of newborns.^(10,11)^ The use of self-inflating bag with the positive end-expiratory pressure (PEEP) valve is desirable for preventing and minimizing the deleterious effects caused by disconnecting the patient from the mechanical ventilator.^(12,13)^

The expansion of collapsed areas and the removal of peripheral secretions represent the therapeutic effects of these techniques that promote increased lung volume. Such effects can be achieved by reducing the alveolar pressure using either deep inspiration techniques or those that apply positive pressure. In newborns, due to the anatomical and physiological characteristics of their respiratory systems, lung expansion therapy using continuous or intermittent positive airway pressure is adopted.^(14-17)^

Manual hyperinflation is often used in children under invasive mechanical ventilation (IMV) in neonatal units; however, few studies have addressed the effects of this technique on this specific population. The study hypothesis was that the use of a manual resuscitator with the PEEP control valve would be beneficial during MH in preterm newborns undergoing IMV.

The aim of the study was to assess the effects of manual hyperinflation combined with chest vibration performed with a manual resuscitator, with and without the positive end-expiratory pressure control valve, on the respiratory function in preterm newborns undergoing invasive mechanical ventilation.

## METHODS

This prospective cross-sectional study was conducted in Neonatal Intensive Care Center 1 of the *Hospital das Clínicas* of the *Faculdade de Medicina* of the *Universidade de São Paulo* (USP), from January 2010 to December 2012. The number of admissions to the nursery during this period was 5,572 newborns, 1,838 of whom were preterm. The project was approved by the Ethics Committee for the Analysis of Research Projects of the institution (no. 0027/10).

A total of 28 preterm newborns met the inclusion criteria of gestational age at birth of less than 32 weeks, undergoing IMV for more than 14 consecutive days and dependent on it at 28 days of life. All patients presented bronchial hypersecretion or radiological evidence by indication of the medical team and were hemodynamically stable (without the use of vasoactive drugs), with systemic blood pressure within the normal range for their gestational age and with good global perfusion.^(18)^

**Figure 1:**
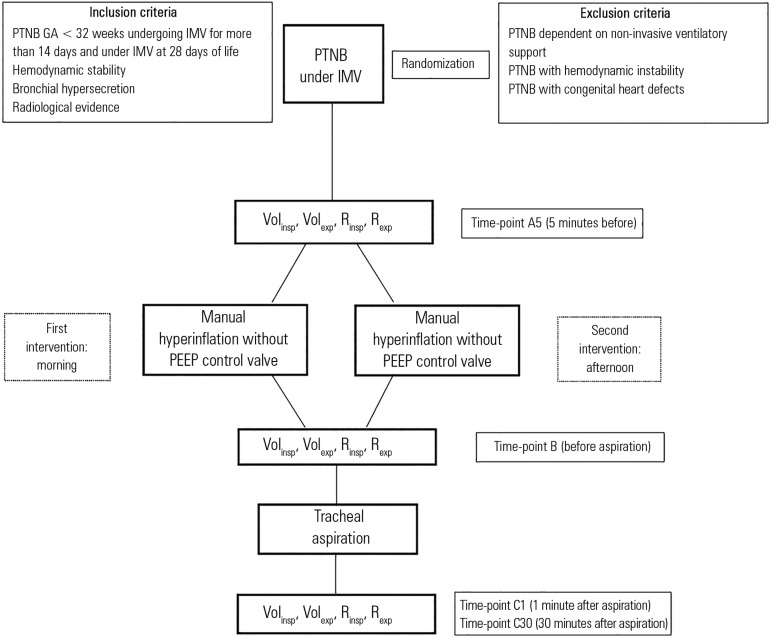
Study design. PTNB - preterm newborn; GA - gestational age; IMV - invasive mechanical ventilation; PEEP - positive end- pressure; vol_insp_ - inspiratory volume; vol_exp_ - expiratory volume; r_insp_ - inspiratory resistance; r_exp_ - expiratory resistance.

Preterm newborns dependent on non-invasive respiratory support, newborns with hemodynamic instability, and those with congenital heart defects were excluded. Through review of the medical records, a database was created for each patient containing the following information: gender, gestational age at birth, birth weight, nutritional adequacy, 1- and 5-minute APGAR scores, diagnosis, current weight and corrected gestational age. Following authorization by the parents or guardians, through the signing of an informed consent form, preterm newborns under mechanical ventilation were selected based on the inclusion criteria (Figure 1).

Manual hyperinflation was applied to all patients with a silicone manual resuscitator (HSiner Newmed^®^, with a 500mL capacity and a spring-loaded PEEP control valve spring, alternating the use or not of the valve.

The MH technique was applied to the newborns using a manual resuscitator with and without the PEEP control valve to assess the effects of the maneuver under both conditions. The PEEP value during MH was the same as that received by the patient when connected to the ventilator. An oxygen flow rate of 5L/minute (the routine procedure within the unit) was used during the maneuver. The inspiratory pressure employed during the intervention was controlled based on the lung expansion of the newborn through direct observation of chest movement between 0.5 and 1.0cm.

Manual hyperinflation was performed with six consecutive slow and deep inspirations, followed by a 2-second inspiratory pause and a quick pressure release, combined with chest vibration, thus promoting increased expiratory flow, according to the standard operating procedure of the physical therapy service of the institution where the study was conducted. After MH combined with chest vibration, tracheal aspiration was performed, thus ending the maneuver.

The variables studied were inspiratory and expiratory lung volumes and inspiratory and expiratory pulmonary resistance, measured using an Inter GMX Slim-Intermediate^®^ graphic breathing monitor.

The variables were analyzed at the following time-points: 5 minutes before the maneuver (time-point A5), immediately after the maneuver (time-point B), 1 minute after aspiration (time-point C1), and 30 minutes after aspiration (time-point C30). For each variable studied, three measurements were recorded at the analyzed time-points, and the maximum measurement of each variable was considered in the analysis.

All patients included in the study were ventilated using the same mechanical ventilator as the one used in the neonatal unit where the data were collected. A continuous-flow, pressure-limited and time-cycled Interneo mechanical ventilator (Intermed^®^) was used in synchronized intermittent mandatory ventilation (SIMV) + pressure support (PS) mode. This ventilator has a proximal flow sensor (pneumotachograph) that records variations in pulmonary parameters every 25ms using the Inter GMX Slim-Intermediate^®^ graphic breathing monitor; the ventilatory parameters were adjusted according to the patient's clinical condition and gasometric and X-ray tests, in accordance with the unit's routine procedures.

The variables were controlled in two respiratory physical therapy interventions, one in the morning and another in the afternoon, and the maneuver was performed with and without the PEEP control valve in the same patient. That is, each newborn included in the study received an intervention with the PEEP valve and another without the valve, according to the randomization performed through drawing lots at the time of the newborn's inclusion in the study. The newborn remained under treatment until resolution of the bronchial hypersecretion and/or radiological improvement.

The intervention in the newborn was interrupted if peripheral oxygen saturation dropped below 85% or if their heart rate increased or decreased to values above 170 or below 100 beats per minute, respectively, which are signs of increased respiratory effort with retractions.

The PEEP value was adjusted using a pressure gauge attached to the MH with 5L/minute oxygen flow. The respiratory physical therapy intervention was conducted by the responsible investigator and five specialized physical therapists of the Physical Therapy team at the *Instituto da Criança*, who had an average experience of 15.5 ± 3.93 years, with a median of 15 years, and had been previously trained on the maneuver studied. The variables were recorded by a professional from the multidisciplinary team (not the physical therapist), who was blinded to the resource used.

For sample size calculations, the percent changes of each variable evaluated between time-points A5 and B and B and C1 were considered, relative to their respective values in the initial time-points instead of their absolute values. The time-points A5 and B and B and C1 were considered for the sample calculation. In time-points B and C1, for the variable peripheral oxygen saturation, a reduction of 10% was expected, and a reduction or increase of 3% was defined as a clinically significant difference. For this purpose, the two-tailed Wilcoxon test was used at a 5% significance level and 80% power. The calculated sample size was 28 patients. Given the study design, where MH with and without the valve was performed sequentially in the same newborns, mixed models were used to compare the procedures.

## RESULTS

A total of 28 newborns were included in the study. In all patients, two respiratory physical therapy interventions were performed using MH with and without the PEEP control valve. The characteristics of the studied population are summarized in table 1.

**Table 1:** Characteristics of the patients studied (n=28)

Characteristics	
Gestational age at birth (weeks)	28.90 ± 1.79
Birth weight (g)	1,005.71 ± 372.16
Sex	
Male	17 (60, 7)
Female	11 (39, 3)
Nutritional adequacy	
Appropriate for gestational age	11 (39, 3)
Small for gestational age	17 (60, 7)
Large for gestational age	0 (0)
Corrected gestational age (weeks)	33.26 ± 1.78
Weight day (g)	1,296.61 ± 352.46
Diagnostics (respiratory diseases)	
Bronchopulmonary dysplasia	28 (100)
Time on mechanical ventilation (days)	29.5 (15-53)

Results expressed by number (%), average ± standard deviation and median (25% - 75%).

All newborns were ventilated in SIMV + PS mode. The mechanical ventilation parameters showed no statistically significant differences in any of the studied time-points regardless of the use or not of the PEEP control valve. The fraction of inspired oxygen (%) presented p = 0.774, breathing rate (bpm) p = 0.970, peak inspiratory pressure (PIP) (cmH_2_O) p = 0.984, PEEP (cmH_2_O) p = 0.978, flow rate (L/min) p = 0.973, PS (cmH_2_O) p = 0.974, mean arterial pressure (MAP) (cmH_2_O) p = 0.864 and inspiration time (seconds) p = 0.983.

The results regarding pulmonary function are presented in table 2, and the comparison of variables between the studied time-points is summarized in table 3.

**Table 2:** Measurements of the studied variables according to time-point and intervention with and without the positive end-expiratory pressure valve

	NVA5	NVB	NVC1	NVC30	WVA5	WVB	WVC1	WVC30
Inspiratory volume (mL)	8.52 ± 4.67	9.13 ± 5.1	10.03 ± 5.79	9.38 ± 4.7	9.76 ± 4.4	9.67 ± 6.4	10.83 ± 6.38	9.7 ± 4.14
Expiratory volume (mL)	9.21 ± 5.17	10.08 ± 6.47	12.47 ± 8.76	9.98 ± 5.23	9.92 ± 4.64	9.84 ± 5.9	11.71 ± 6.85	11.05 ± 6.63
Inspiratory resistance (hpa/L/s)	105.18 ± 49.83	95.36 ± 55.58	86.46 ± 53.73	84.86 ± 52.43	95.46 ± 61.29	97.93 ± 57.69	91.43 ± 63.89	80.14 ± 46.07
Expiratory resistance (hpa/L/s)	29.14 ± 29.27	36.68 ± 33.46	41.11 ± 38.01	25.21 ± 31.41	23.29 ± 18.55	36.18 ± 42.3	32.39 ± 33.45	23.43 ± 19.84

NVA5 - without valve time-point A5; NVB - without valve time-point B; NVC1 - without valve time-point C1; NVC30 - without valve time-point C30; WVA5 - with valve time-point A5; WVB - with valve time-point B; WVC1 - with valve time-point C1; WVC30 - with valve time-point C30.

**Table 3:** Comparison of variables between the time-points without and with the valve

Time-points	p value
Inspiratory volume (mL)	
A5 *versus* C1 - NV	0.026
A5 *versus* B - NV	0.365
A5 *versus* C30 - NV	0.202
Expiratory volume (mL)	
A5 *versus* C1-NV	0.001
A5 *versus* B - NV	0.358
A5 *versus* C30 - NV	0.416
Inspiratory resistance (hpa/L/s)	
A5 *versus* B - NV	0.396
B *versus* C1 - NV	0.442
C1 *versus* C30 - NV	0.889
A5 *versus* C30 - NV	0.08
Expiratory resistance (hpa/L/s)	
A5 *versus* B - NV	0.2122
B *versus* C1 - NV	0.4629
A5 *versus* C30 - NV	0.5148
Inspiratory volume (mL)	
A5 *versus* C1-WV	0.001
A5 *versus* B - WV	0.89
A5 *versus* C30 - WV	0.928
C1 - SV *versus* WV	0.237
Expiratory volume (mL)	
A5 *versus* C1 - WV	0.009
A5 *versus* B - WV	0.931
A5 *versus* C30 - WV	0.236
C1 - SV *versus* WV	0.422
Inspiratory resistance (hpa/L/s)	
A5 *versus* B - WV	0.831
B *versus* C1 - WV	0.574
C1 *versus* C30 - WV	0.33
A5 *versus* C30 - WV	0.186
B *versus* C1 - WV	0.5302
Expiratory resistance (hpa/L/s)	
C1 *versus* C30 - WV	0.1382
A5 *versus* C30 - WV	0.9811

A5 - immediately before tracheal aspiration; C1 - 1 minute after aspiration; NV - without valve; B - Immediately after the maneuver; C30 - 30 minutes after tracheal aspiration; WV - with valve.

## DISCUSSION

In patients under mechanical ventilation, the use of PEEP increases the functional residual capacity, preventing alveolar collapse and injury by cyclic stretching, thus minimizing inflammation arising from the recurrent opening and closing of the airways.^(19-21)^ Recently, a modification of the manual hyperinflation technique, known as the mechanical ventilator hyperinflation, was developed with the aim of avoiding possible adverse effects resulting from disconnecting the patient from respiratory support and the withdrawal of PEEP.^(22)^ Some authors have demonstrated that this maneuver, associated with PEEP, leads to mobilization of secretions in the lower airways and increased lung compliance. With the use of higher pressures and tidal volumes than those defined for ventilation, clearing of the lower airways occurs, with improved ventilation in areas previously little- or non-ventilated.^(23-25)^

Manual hyperinflation has limitations, such as the deleterious effect inherent to disconnecting the patient from the mechanical ventilator and the reduced control over mean pressure, tidal volume, flow rate, fraction of inspired oxygen and pressure limit.^(23-25)^ The disconnection from the mechanical ventilator and, consequently, the withdrawal of PEEP can mainly cause shearing injury related to the cyclic opening and closing of unstable lung units.^(26,27)^

Analyzing tidal volume, we observed an increase as of time-point B, in maneuvers both with and without the valve, with the largest volumes in time-point C1. Descriptively, the mean values of inspiratory volume did not return to their initial levels, with apparently constant variations at all time-points. The expiratory volume increased as of time-point B, with the largest volumes being observed at time-point C1. Descriptively, the mean values did not return to the initial levels, and the variation was apparently constant at all time-points. There was no significant difference in the expiratory volume between time-points in the maneuvers with and without the valve. Analyzing the behavior of the inspiratory and expiratory volumes between time-points A5 and B and time-points A5 and C30, we found no significant difference between the means in maneuvers with and without the valve.

We observed that the inspiratory and expiratory volumes increased in both maneuvers but without a significant difference, except for time-point C1. This trend suggests that the MH maneuver is beneficial to the patient, increasing lung volumes, regardless of the use of the valve. However, this result may have been influenced by the small variation observed due to the small sample size.

There was a decrease in inspiratory resistance between time-points A5 and C30 for each maneuver, and the mean values did not return to their initial levels, with apparently constant variation at all time-points. The significant decrease in inspiratory resistance between the initial time-point and after the interventions can be attributed to the beneficial effect of mobilization, the removal of secretions and the increased functional residual capacity increase. We concluded that the use of the PEEP valve reinforces these effects, resulting in a sharper decline in inspiratory resistance. The lack of a significant difference in inspiratory resistance between the time-points within each maneuver could be due to the sample size.

With respect to the expiratory resistance, for maneuvers without the valve, there was an increase in this parameter at time-points B and C1, with a return to the initial level at C30. For maneuvers with the valve, the expiratory resistance value at C1 was already smaller than at B; that is, the return of the increase in mean expiratory resistance to the initial level occurred more quickly in the presence of the valve. This result shows that the increase in expiratory resistance was less pronounced when using the valve. In the comparison between time-points, there was a significant difference only between time-points C1 and C30 for maneuvers without the valve and between time-points A5 and B for maneuvers with the valve. In the first condition, the results can be explained by the variation in the sample. With respect to time-points A5 and B for maneuvers with the valve, it is known that when using PEEP devices, the resistance to the expiratory circuit is primarily attributed to the resistance of this valve. However, patients under mechanical ventilation can present active expiratory effort. In addition, spring-loaded valves generate pressure, and when there is increased expiratory flow, such as with a cough or during MH, high pressures can occur in the respiratory system. In this situation of increased forced expiratory volume, expiration is no longer passive, and increases in expiratory effort, oxygen consumption and expiratory resistance occur.^(28)^

One of the limiting factors of the study was not using pressure gauges to measure and control peak inspiratory pressure. Thus, lung expansion was controlled by observing chest expansion between 0.5 and 1.0cm, which may have contributed to variations in the collected data. Another limiting factor was the heterogeneity of the sample regarding the mechanical ventilation time, with different degrees of maturity and lung injury, which could have interfered with the results, not allowing the identification of differences in treatments with and without PEEP valves.

Overall, the study did not allow statistical differentiation between the effects of MH with and without the PEEP control valve on respiratory function in preterm newborns under prolonged mechanical ventilation. However, it was possible to find clinical evidence of the beneficial effects of the PEEP valve, such as increased lung volumes and decreased pulmonary resistance. Additional studies with larger samples are recommended.

## CONCLUSION

The inspiratory and expiratory volumes increased in both maneuvers, with a significant difference in the first minute after tracheal aspiration. This trend suggests that the manual hyperinflation technique is beneficial for the patient, increasing lung volumes, regardless of the use of the positive end-expiratory pressure valve.

There was no significant difference in the inspiratory resistance between time-points within each maneuver. There were significant increases in expiratory resistance in the comparison between the first minute and 30 minutes after the end of maneuvers without the valve and between 5 minutes before and immediately before tracheal aspiration for maneuvers with the valve. This result can be attributed to the variation in the sample in the first situation and to the physiological effects of positive end-expiratory pressure in maneuvers with the valve.
